# Molecular Characterization of African Swine Fever Virus, China, 2018

**DOI:** 10.3201/eid2411.181274

**Published:** 2018-11

**Authors:** Shengqiang Ge, Jinming Li, Xiaoxu Fan, Fuxiao Liu, Lin Li, Qinghua Wang, Weijie Ren, Jingyue Bao, Chunju Liu, Hua Wang, Yutian Liu, Yongqiang Zhang, Tiangang Xu, Xiaodong Wu, Zhiliang Wang

**Affiliations:** China Animal Health and Epidemiology Center, Qingdao, China

**Keywords:** African swine fever, ASF, African swine fever virus, ASFV, viruses, molecular characterization, genotype, serotype, causative virus strain, outbreak, pigs, zoonoses, China

## Abstract

On August 3, 2018, an outbreak of African swine fever in pigs was reported in China. We subjected a virus from an African swine fever–positive pig sample to phylogenetic analysis. This analysis showed that the causative strain belonged to the p72 genotype II and CD2v serogroup 8.

African swine fever (ASF) is a disease that is reportable to the World Health Organisation for Animal Health. This disease causes high fever, hemorrhages, ataxia, and severe depression in domestic pigs and has mortality rates approaching 100%. Its causative agent is African swine fever virus (ASFV; family *Asfarviridae*, genus *Asfivirus*), a large, enveloped, double-stranded DNA virus (*1*). ASF was first described in Kenya in 1921, and was introduced into the Republic of Georgia in 2007, after which it spread into other countries in eastern Europe, including Russia (2007), Ukraine (2012), Belarus (2013), Lithuania (2014), Estonia (2014), Poland (2014), Latvia (2014), Romania (2017), the Czech Republic (2017), and Hungary (2018).

During July 1–August 1, 2018, a total of 47 of 383 pigs died on a farm in the Shenbei District of Shenyang, Liaoning Province, China. Postmortem analysis performed by local veterinarians showed an ASF-typical lesion in pig spleens that were extremely swollen and severely necrotic. Other pathologic changes included hemorrhages in tonsils and lungs, marbled lesions in mandibular and mesenteric lymph nodes, and diffuse hemorrhages in a large part of gastric serosa.

We collected samples from 2 dead pigs and 6 live pigs on this farm and sent these samples to our Biosafety Level 3 laboratory for confirmation of ASFV infection. We performed a real-time PCR for ASFV as recommended by the World Health Organisation for Animal Health protocol. Results confirmed ASFV infections in China (*2*).

After confirmation of ASFV infection by our laboratory, we used nucleic acid extracts from an ASFV-infected sample for conventional PCR amplification with 3 pairs of primers. We amplified 3 gene fragments: a partial gene fragment of the B646L gene encoding the p72 capsid protein (*3*), a fragment of the EP402R gene encoding the CD2v protein (*4*), and a tandem repeat sequence (TRS) located between the I73R and I329L genes (*5*).

We subjected 3 amplified products to nucleotide sequencing and deposited the resulting sequences in GenBank (accession nos. MH722357, MH735142, and MH735144). We used the p72 fragment sequence for phylogenetic analysis of the genotype, and the CD2v fragment sequence for phylogenetic analysis of the serogroup (*6*). We constructed 2 phylogenetic trees by using MEGA 5.0 software (https://www.megasoftware.net/). These trees showed that the causative strain (China 2018/1) in this study belonged to p72 genotype II (Figure, panel A) and to CD2v serogroup 8 (Figure, panel B).

**Figure:**
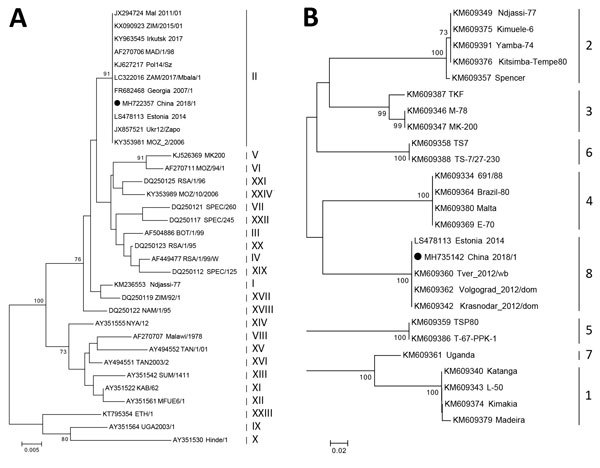
Phylogenetic analysis of a causative virus strain (China 2018/1) of an African swine fever outbreak, China, 2018. A) p72 genotype; B) CD2v serogroup. The neighbor-joining method and Kimura 2-parameter model were used for construction of phylogenetic trees in MEGA 5.0 software (https://www.megasoftware.net/). Numbers along branches indicate bootstrap values >70% (1,000 replicates). Black circles indicate causative virus from this study. Roman numerals to the right in panel A indicate p72 genotypes. Numbers to the right in panel B indicate CD2v serogroups. GenBank accession numbers are provided for all sequences. Scale bars indicate nucleotide substitutions per site.

Genotype identification of ASFV often depends on partial p72 gene characterization (*3*). During ASF outbreaks, this genotyping approach can be used to identify possible origins of viruses and differentiate them from closely related strains (*7*). In this study, we classified China 2018/1 as genotype II (Figure, panel A), the sequences we obtained had extremely high homology with those of other genotype II strains, therefore suggesting the origin of China 2018/1 from a homogenotypic strain.

In addition to conventional genetic typing, serologic typing is another method for classifying ASFVs on the basis of hemadsorption inhibition (HAI). Eight ASFV serogroups have been identified (*6*). Moreover, HAI typing places ASFV into discrete serogroups not necessarily resolved by the p72-based genetic typing. For example, serogroup 1, 2, and 4 strains can be simultaneously classified as having the P72 genotype I (*7*). In this study, we found that China 2018/1 belonged to serogroup 8 as determined by phylogenetic analysis, suggesting the same HAI characteristics as those for other strains in the homoserogroup (Figure, panel B).

We compared Georgia 2007/1, which is representative of genotype II, with China 2018/1. China 2018/1 had a 10-bp additional fragment (5′-GGAATATATA-3′) that was inserted into the TRS between the I73R and I329L genes and was identical to those of the Bel13/Grodno, Ukr12/Zapo, Lt14/1490, Lt14/1482, Pol14/Sz, and Pol14/Krus strains (*5*).

ASF causes devastating socioeconomic consequences in the global pig industry, especially for countries with large-scale pig production and pork consumption. After the confirmation of ASF outbreak in China in August 2018, we characterized the causative strain, China 2018/1, by phylogenetic comparison with previous strains. We classified this new strain as having the p72 genotype II and 100% p72 sequence identity with several strains from eastern Europe and Africa, such as Bel13/Grodno, Voronezh 2016, Mal 2011/01, and ZIM/2015/01.

On the basis of serologic typing, we found that China 2018/1 belonged to the CD2v serogroup 8. In addition, this strain had a 10-bp additional fragment (5′-GGAATATATA-3′) in its TRS when compared with several others in the homogenotype.

Because ASFV is a large DNA virus with a stable genome, it is hardly unexpected that the sequence of China 2018/1 had extremely high homology with sequences in genotype II over a wide geographic area. However, it remains to be elucidated from where China 2018/1 was introduced into China.
